# Patterns of aeroallergen sensitization in asthma patients identified by latent class analysis: A cross‐sectional study in China

**DOI:** 10.1002/clt2.12271

**Published:** 2023-07-01

**Authors:** Jiale Zhang, Wenting Luo, Guoping Li, Huali Ren, Jie Su, Jianxin Sun, Ruifen Zhong, Siqin Wang, Zhen'an Li, Yan Zhao, Huashou Ke, Ting Chen, Chun Xv, Zhenglin Chang, Liting Wu, Xianhui Zheng, Miaoyuan Xv, Qingyuan Ye, Chuangli Hao, Baoqing Sun

**Affiliations:** ^1^ Department of Clinical Laboratory National Center for Respiratory Medicine, National Clinical Research Center for Respiratory Disease, State Key Laboratory of Respiratory Disease, Guangzhou Institute of Respiratory Health, The First Affiliated Hospital of Guangzhou Medical University Guangzhou China; ^2^ Laboratory of Allergy and Precision Medicine Department of Pulmonary and Critical Care Medicine Chengdu Institute of Respiratory Health Chengdu Third People's Hospital Branch of National Clinical Research Center for Respiratory Disease Chengdu China; ^3^ Department of Allergy State Grid Beijing Electric Power Hospital Capital Medical University Electric Power Teaching Hospital Beijing China; ^4^ The Second People's Hospital of Foshan Foshan China; ^5^ The Second People's Hospital of Zhaoqing Zhaoqing China; ^6^ Dongguan Eighth People's Hospital Dongguan China; ^7^ Henan Provincial People's Hospital Zhengzhou China; ^8^ Foshan Maternal Child Health Hospital Foshan China; ^9^ Department of Allergy The First Affiliated Hospital Harbin Medical University Harbin China; ^10^ Maoming Maternal and Child Health Hospital Maoming China; ^11^ Shengli Clinical Medical College of Fujian Medical University, Department of Otorhinolaryngology Head and Neck Surgery, Fujian Provincial Hospital Fuzhou China; ^12^ Jiangxi Medical College Shangrao China; ^13^ Department of Respiratory Medicine Children's Hospital of Soochow University Suzhou China

**Keywords:** airborne allergen, asthma, latent class analysis (LCA), sensitization pattern, sIgE

## Abstract

**Background:**

This cross‐sectional study aimed to identify latent sensitization profiles of asthma patients in mainland China, unveiling the association between regional differences and sensitization patterns.

**Methods:**

1056 asthma participants from 10 medical centers divided into eastern and western cohorts were clustered into four individual sensitization patterns, respectively, by using an unsupervised statistical modeling method, latent class analysis (LCA), based on the levels of 12 aeroallergens specific IgE reactivities. Moreover, differences in clinical characteristics and environmental exposures were compared in different sensitization patterns.

**Results:**

Four distinct sensitization patterns in the two cohorts were defined as follows, respectively. Eastern cohort: Class 1: “High weed pollen and house dust mites (HDMs) sensitization” (8.87%), Class 2: “HDMs dominated sensitization” (38.38%), Class 3: “High HDMs and animal dander sensitization” (6.95%), Class 4: “Low/no aeroallergen sensitization” (45.80%). Western cohort: Class 1: “High weed pollen sensitization” (26.14%), Class 2: “High multi‐pollen sensitization” (15.02%), Class 3: “HDMs‐dominated sensitization” (10.33%), Class 4: “Low/no aeroallergen sensitization” (48.51%). Of note, the significant statistical difference in age, asthma control test score (ACT) and comorbidities were observed within or between different sensitization patterns. Exposure factors in different sensitization patterns were pointed out.

**Conclusions:**

Asthmatic patients with distinct sensitization patterns were clustered and identified through the LCA method, disclosing the relationship between sensitization profiles of multiple aeroallergens and geographical differences, providing novel insights and potential strategies for atopic disease monitoring, management and prevention in clinical practice.

## 1 INTRODUCTION

Globally, about 300 million patients were affected by asthma currently,^1^ which was one of the most common inflammatory disorders of the airways worldwide. Roughly estimated, the prevalence of asthma in people over 20 years was 4.26% in China, reaching 45.7 million in total, posing a tremendous social and economic burden.^2^

As a primary causative factor, exposure and sensitization to aeroallergens are responsible for most of the onset and persistence of asthma to a large extent.^3^ Alternations have been observed in patterns of aeroallergens sensitization in China in the last decade, demonstrating that geographical differences affect asthmatic patients sensitized.^4^ Besides, previous studies focused more on the northern or southern coastal areas and school‐age children,^5^ while little is known about the differences in aeroallergens sensitization patterns in the western and southwestern regions in mainland China and lack large‐scale epidemiology research in different age periods. Furthermore, quite a part of asthma patients is ignorant of themselves having co‐sensitization to multiple allergens, thus making it more difficult to control and manage in clinical practice.^6^ Conventionally, sensitization patterns of asthmatic patients were defined based on the presence or absence of sensitization to a panel of allergens or the overall number of sensitized allergens in a screening combo, leading to ignorance of the individual heterogeneity and variability of the polysensitization to multi‐allergens.

More recently, latent class analysis (LCA) as an unsupervised statistical modeling method has been widely and successfully used in the identification of phenotypes in multiple chronic respiratory diseases. Latent class analysis is to explain the correlation among different observed variables with the least latent variables, satisfying the requirement of local independence between observed variables within each latent category meanwhile.^7^ Then, all subjects would be clustered and divided into different latent classes, by calculating and comparing the biggest posterior conditional probability. Concerning a latent variable, the larger the conditional probability, the greater the weight, indicating the latent variable showed a strong influence on the observed variable in the latent category. Compared with traditional hierarchical cluster analysis (HCA), the essence of LCA is to discover potential variables, regarded as a combination of principal component analysis (PCA) and cluster analysis, providing a more integral view of detailed information reflected by explicit variables without eliminating variables and reducing people's intervention.

Therefore, the aim of this multicenter study was first to establish a flexible statistical model for clustering asthma patients from eastern and western regions of China mainland into distinct sensitization patterns through the LCA method respectively. Secondly, identify and verify differences in clinical characteristics among different latent categories in two cohorts based on levels of 12 aeroallergens specific IgE. Finally, explore possible risk factors in different cluster populations by using multivariate logistic regression, providing new insights and strategies in the clinical management and precision diagnosis of atopic diseases.

## 2 MATERIALS AND METHODS

### 2.1 Patients and study design

**FIGURE 1:**
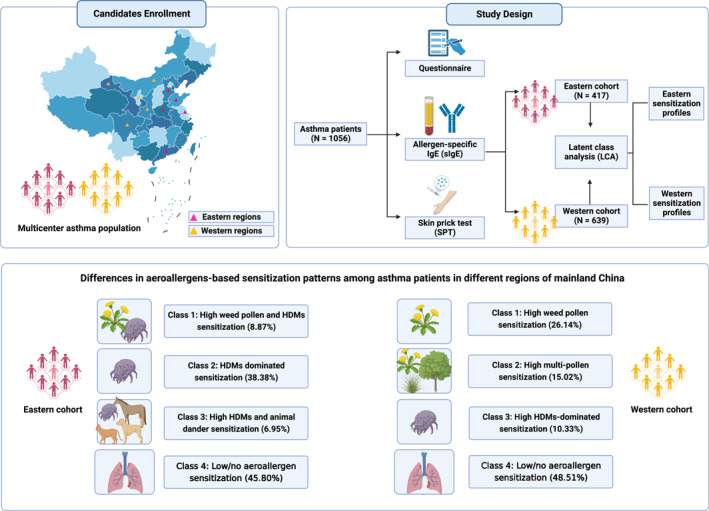
Graphical abstract of this cross‐sectional study.

This cross‐sectional study was conducted in 10 medical centers in China between September 2021 and October 2022, including 5 eastern regions (Jiangsu, Shandong, Guangdong, Henan, and Hebei) and 5 western regions (Gansu, Sichuan, Ningxia, Inner Mongolia, and Shaanxi). Patients can be diagnosed as asthma if he/she meets the following symptoms and meets any of the objective tests for airflow limitation, except wheezing, shortness of breath, chest tightness and cough caused by other diseases: (a) recurrent wheezing, shortness of breath, with or without chest tightness or cough, frequent at night and in the morning; (b) sporadic or diffuse wheezing sounds could be heard in both lungs; (c) objective inspection of variable airflow limitation: bronchodilation test was positive, positive bronchial excitation test, peak expiratory flow (PEF) was >10%, or the weekly variation rate of PEF was >20%. Patients who at least sensitized to one of the allergens in Skin prick test (SPT) (Grade 1–4) or sIgE detection (>0.35 kU/L) will be diagnosed as allergic asthma (AA). After excluding the missing data, 1056 asthmatic patients aged 0–86 years were recruited, according to the guidelines of the Global Initiative for Asthma (GINA)^8^ and Chinese.^9^ After signing informed consent, patients underwent an SPT with 16 aeroallergens and collected 5 mL venous blood for serum‐sIgE detection of 12 aeroallergens. Furthermore, patients and/or their legal guardians filled in a questionnaire. The study design was presented in Figure 1 and created with BioRender.com.

### 2.2 Standardized questionnaire

The questionnaire consisted of basic information, clinical history, ACT score and environmental exposures. Demographic characteristics included age, gender, ethnic group and delivery mode. Clinical history showed patients' comorbidity situations. Additionally, environmental exposures consisted of living surroundings and the history of exposures. Lastly, the ACT score was calculated after answering five questions about asthma control as listed below.^10^ (i) How often has asthma influenced your daily activities (work/study/rest) in the past four weeks? (ii) How many times have you had difficulty breathing (anhelation, shortness of breath or poor breathing) in the past four weeks? (iii) How many times have you woken up at night or earlier than usual in the morning due to asthma symptoms (wheezing, coughing, dyspnea, chest tightness or pain), in the past four weeks? (iv) How many times have you used emergency medicine (such as salbutamol) in the past four weeks? (v) How did you assess your asthma control over the past four weeks? According to GINA criteria, the ACT score was classified into three different groups: (i) ACT score<20, uncontrolled; (ii) 20 = ACT score<25, partially controlled; and (iii) ACT score≥25, controlled. All completed questionnaires were verified and double‐checked by two well‐trained investigators.

### 2.3 Skin prick test (SPT)

All candidates underwent SPT by using commercial extracts (ALK‐Abell´o Lab‐oratory, Madrid, Spain) of 16 common aeroallergens, including 4 indoor allergens (*Dermatophagoides pteronyssinus, Blattella germanica, Aspergillus fumigatus, Penicillium notatum*) and 12 outdoor allergens (*Ambrosia elatior, Artemisia vulgaris, Chenopodium album, Betula verrucosa, Phleum pratense, Ulmus campestris, Salix fragilis, Cynodon dactylon, Populus alba, Mediterranean cypress, Platanus hispanica, Phragmites communis*). Histamine and normal saline serve as positive and negative controls respectively. All performances followed the standard operation procedure. After 15 min, the wheal reaction was measured as the mean of the maximum diameter and the length of the perpendicular line through its middle. Any allergen showing the size of a wheal ≥3 mm than the negative control should be considered a positive reaction. The result was presented as skin index (SI = mean size of allergen wheal/mean size of histamine wheal). SI accounts for 25%, 50%, 100% and 200% of the histamine‐induced wheal area was defined as Grade 1, 2, 3 and 4 respectively. Grades 1–4 were considered as positive skin reactions, whereas Grade 0 was suggested as a negative reaction. All patients enrolled in the study had discontinued antiallergic drugs for at least 14 days before the SPT.

### 2.4 Detection of the level of sera‐specific IgE (sIgE)

Asthma patients were offered blood draw for the detection of 12 aeroallergen IgE, including 6 indoor allergens, D1 (*Dermatophagoides pteronyssinus*), D2 (*Dermatophagoides farinae*), E1 (cat), E3 (horse), E5 (dog) I6 (*Blattella germanica*), and 6 outdoor allergens, W6 (mugwort), W7 (marguerite), W8 (dandelion), W9 (plantain), G6 (timothy), T3 (birch). Serum samples were centrifuged for 10 min at 3000 rpm and maintained at −80°C for long‐term storage. Samples were analyzed with the ALLEOS 2000^TM^ allergen detection system (Hycor Biomedical, USA). Based on RAST classification, levels of sIgE were quantitatively categorized into six classes: Class 0, <0.35 kU/L; Class 1, 0.35–0.70 kU/L; Class 2: 0.70–3.50 kU/L; Class 3: 3.50–17.50 kU/L; Class 4: 17.50–50.00 kU/L; Class 5: 50.00–100.00 kU/L and Class 6: ≥100.00 kU/L.

### 2.5 Latent class analysis (LCA)

Latent class analysis is a classification method of various latent variables by establishing a statistical model to describe the correlation between explicit variables and latent variables. First, the classification models were repeatedly fitted in a stepwise fashion and selected through model comparison based on the optimal model fit parameters (Akaike Information Criterion, AIC; Bayesian Information Criterion, BIC; entropy; Vuong‐Lo‐Mendell‐Rubin Likelihood Ratio, LMR *p*‐value; Parametric Bootstrapped Likelihood Ratio Test, BLRT *p*‐value; Additional File 1 Tables S1‐S2). Second, all subjects were divided into different categories based on conditional probability with post hoc tests. Lastly, summarize and define the characteristics of different sensitization profiles. Each cohort has identified one cluster with low aeroallergen‐sensitized populations for subsequent analysis.

### 2.6 Statistical analysis

Quantitative variables were described as mean and standard deviation (mean ± S.D.), while qualitative variables were expressed as frequency or percentage. Different data were analyzed by one‐way ANOVA, chi‐square test or multiple logistic regression with IBM SPSS v. 22 software (IBM Corp., Armonk, NY, USA), respectively. Figures were plotted by GraphPad Prism v. 7 (San Diego, CA, USA) and R v. 4.2.1(Core Team 2022). Latent class analysis was conducted by Mplus software v.8.3 (Los Angeles, CA). A *p*‐value <0.05 was regarded as statistically significant. Significance is displayed in figures as follows: *, *p* < 0.05; **, *p* < 0.01; and ***, *p* < 0.001.

## 3 RESULTS

### 3.1 Characteristics of study participants

In this multicenter study, 417 and 639 asthmatic patients were recruited from the eastern and western regions of China, respectively. The median age of the patients was 20.89 years 461 (43.66%) and 595 (56.34%) participants were female and male respectively. More specific details were shown in Table 1 and Additional File 1 Table S3.

**TABLE 1:** Baseline characteristics and demographics of asthmatic patients.

Characteristics	Total (*N* = 1056)	Eastern cohort (*N* = 417)	Western cohort (*N* = 639)	*p*‐value
Clinical information				
Age, (mean ± SD)	20.89 ± 19.59	22.93 ± 21.23	19.57 ± 18.36	0.006
Gender, No. (%)				0.238
Male	595(56.34)	226(54.20)	369(57.75)	
Female	461(43.66)	191(45.80)	270(42.25)	
Ethnic group				<0.001
Han Chinese	981(92.90)	412(98.80)	569(89.05)	
China's ethnic minorities	75(7.10)	5(1.20)	70(10.95)	
Delivery mode, No. (%)				0.519
Eutocia	757(71.69)	299(71.70)	458(71.67)	
Cesarean	299(28.31)	118(28.30)	181(28.33)	
Asthma control test score, (mean ± SD), No. (%)	23.17 ± 0.42	23.52 ± 0.72	22.95 ± 0.51	0.505
<20	398(37.69)	164(39.33)	234(36.62)	
20≤ ACT score <25	453(42.90)	153(36.69)	300(47.26)	
≥25	205(19.41)	100(23.98)	105(16.12)	
Allergic diseases				
Asthma, No. (%)				N.A.
Yes	1056(100.00)	417(39.49)	639(60.51)	
Allergic rhinitis (AR), No. (%)				0.133
Yes	867(82.10)	356(85.37)	511(80.22)	
No	189(17.90)	61(14.63)	128(19.78)	
Allergic conjunctivitis (AC), No. (%)				0.917
Yes	505(47.82)	199(47.72)	306(47.89)	
No	551(52.18)	218(52.28)	333(52.11)	
Skin allergy, No. (%)				0.565
Yes	495(46.79)	192(46.04)	303(47.27)	
No	448(42.34)	182(43.65)	266(41.50)	
Unknow	113(10.87)	43(10.31)	70(11.23)	
Food allergy, No. (%)				0.003
Yes	87(8.22)	27(6.47)	60(9.36)	
No	725(68.53)	346(82.97)	379(59.13)	
Unknow	244(23.25)	44(10.56)	200(31.51)	
Environmental exposures				
Trees, grass or flowers exposure, No. (%)				<0.001
Symptoms appear after exposure	287(27.17)	75(17.99)	212(33.18)	
Without symptoms after exposure	591(55.97)	247(59.23)	344(53.83)	
No exposure	178(16.86)	95(22.78)	83(12.99)	
Furry animal exposure, No. (%)				<0.001
Symptoms appear after exposure	110(10.42)	23(5.52)	87(13.62)	
Without symptoms after exposure	338(32.01)	138(33.09)	200(31.30)	
No exposure	608(57.57)	256(61.39)	352(55.08)	
Location of residence, No. (%)				0.006
Urban	932(88.26)	354(84.89)	578(90.45)	
Rural	124(11.74)	63(15.11)	61(9.55)	
Domestic storey, No. (%)				0.691
<9	373(35.32)	144(34.53)	229(35.84)	
≥9	683(64.68)	273(65.47)	410(64.16)	
Tobacco smoke exposure, No. (%)				0.291
Yes	547(51.80)	208(49.88)	339(53.05)	
No	509(48.20)	209(50.12)	300(46.95)	
Using mattress, No. (%)				0.383
Yes	945(89.49)	369(88.49)	576(90.14)	
No	111(10.51)	48(11.51)	63(9.86)	
Type of quilts, No. (%)				0.001
cotton	835(78.92)	319(76.50)	516(80.50)	
others	330(31.19)	162(38.85)	168(26.21)	
Using air conditioners, No. (%)				<0.001
Yes	696(65.91)	390(93.53)	306(47.89)	
No	360(34.09)	27(6.47)	333(52.11)	

### 3.2 Sensitization rates and distributions of 16 representative aeroallergens on SPT

**FIGURE 2:**
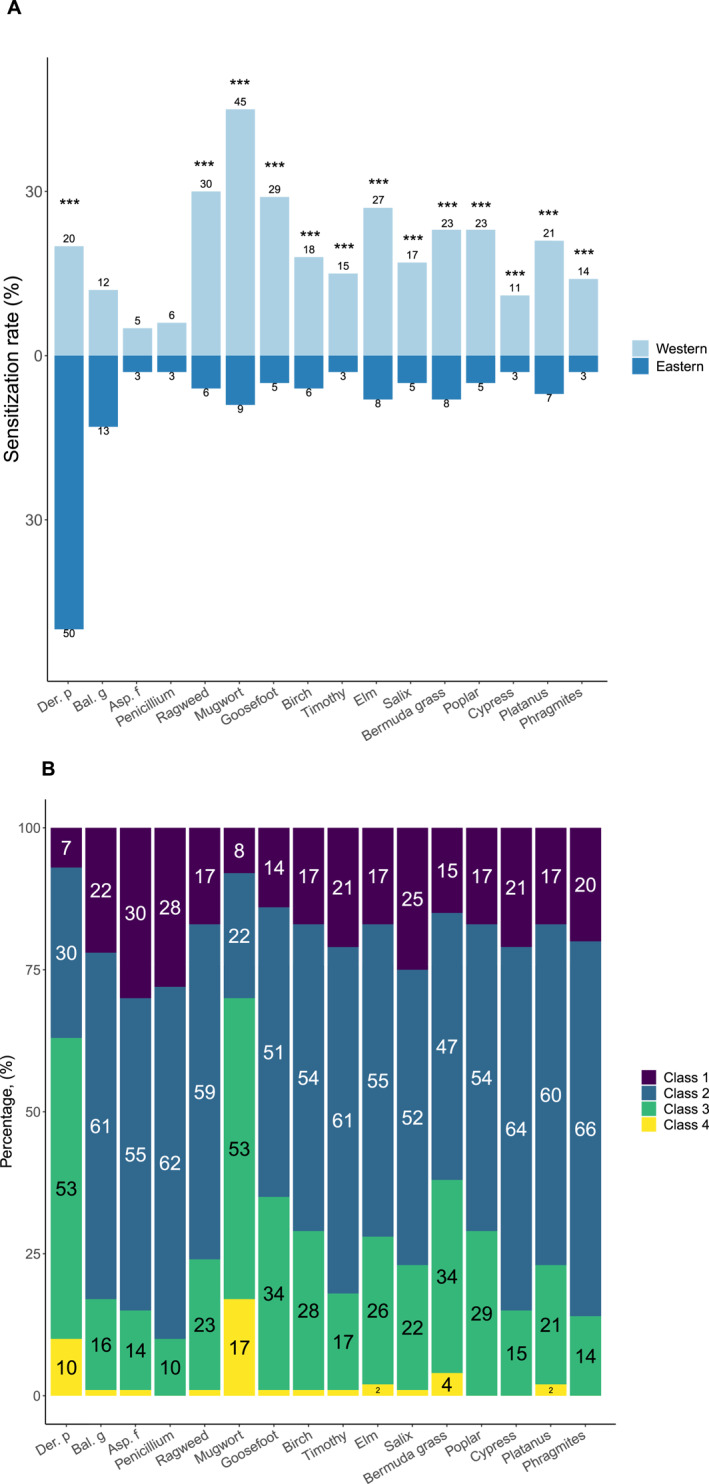
Sensitization rates (A) and distributions (B) of 16 aeroallergens on Skin prick test (SPT) in two regions. The differences in sensitization rates of different aeroallergens were compared between the two cohorts (*, *p* < 0.05; **, *p* < 0.01; and ***, *p* < 0.001). Der. p (*Dermatophagoides pteronyssinus*), Bla. g (*Blattella germanica*), Asp. f (Aspergillus fumigatus).

Remarkably, a high sensitization rate to house dust mites (HDMs) of 50.0% was noted in patients in the eastern regions, followed by cockroaches (13.0%), while sensitization rates of other allergens were below 10.0%. On the contrary, SPT results in western areas showed that 45.0% of patients were sensitization to mugwort, followed by ragweed (30.0%), goosefoot (30.0%), elm (29.0%), poplar (23.0%) and Bermuda grass (23.0%), demonstrating that the western cohort featured in sensitization to multi‐pollen with an increasing sensitization rate of HDMs (20.0%) (Figure 2A). Specifically, over half of the cases developed grade 2 reactions on SPT in 13 allergens, except *Der. p* and mugwort were mainly developed grade 3 reactions (Figure 2B). The difference in sensitization rates of different aeroallergens between the two cohorts was statistically significant (***, *p* < 0.001).

### 3.3 Classification and characteristics of sensitization patterns in different cohorts

**FIGURE 3:**
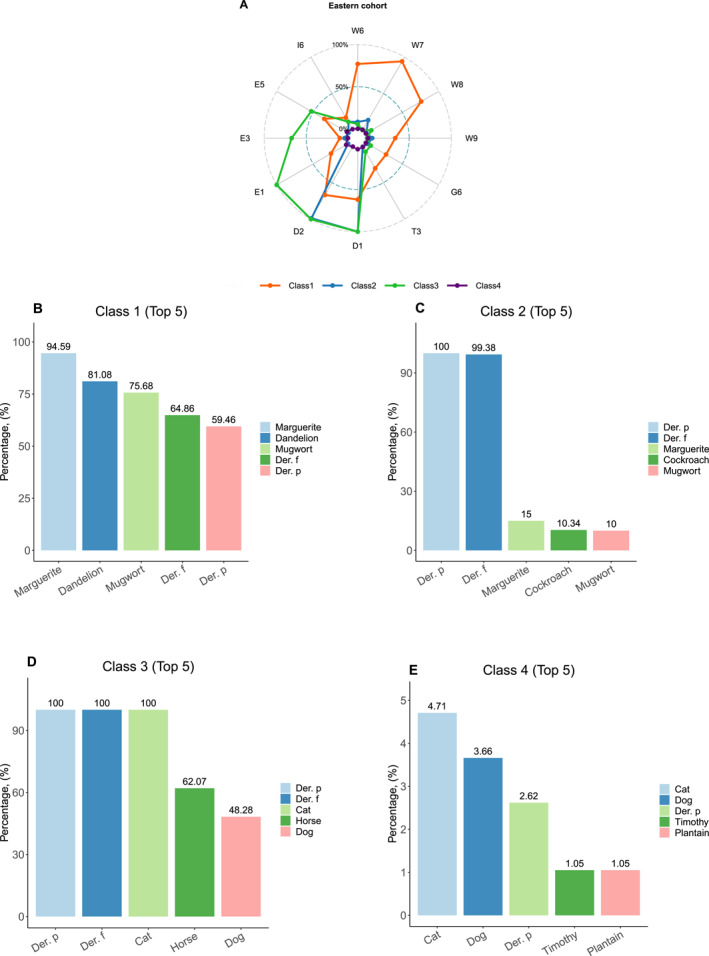
Differences in 12 aeroallergens sensitization patterns. (A) based on sIgE reactivity (B‐E) in the eastern cohort. In the eastern cohort, the radar plots showed the sensitization rate of each aeroallergen in different sensitization patterns with the posterior probability (Figure 3A). While the bar charts demonstrated the top 5 sensitization rates of 12 aeroallergens in different sensitization patterns (Figure 3B–E). 12 aeroallergens included W6 (mugwort), W7 (marguerite), W8 (dandelion), W9 (plantain), G6 (timothy), T3 (birch), D1 (*Dermatophagoides pteronyssinus*), D2 (*Dermatophagoides farinae*), E1 (cat), E3 (horse), E5 (dog) and I6 (*Blattella germanica*).

In the eastern cohort, 417 participants were clustered into four distinct sensitization categories using the LCA model. Four classes were defined as follows. Class 1: “High weed pollen and HDMs sensitization” (*n* = 37 [8.87%]), Class 2: “HDMs dominated sensitization” (*n* = 160 [38.38%]), Class 3: “High HDMs and animal dander sensitization” (*n* = 29 [6.95%]), Class 4: “Low/no aeroallergen sensitization” (*n* = 191 [45.80%]) (Figure 3A). Besides, the top 5 sIgE levels of allergen‐specific shown in Figure 3B–E verified the accuracy of sensitization patterns in clustering and classification. Patients in Class 4 were characterized with close to zero probability of sensitization to all 12 aeroallergens. Except for the Class 4 population, half of the patients in the eastern cohort were commonly sensitization to HDMs (Der. p [52.0%], Der. f [51.0%]). Patients in Class 1 exhibited high sensitization to both weed pollen (marguerite [94.59%], dandelion [81.08%], mugwort [75.68%]) and HDMs (Der. f [64.86%], Der. p [59.46%]). Moreover, both Class 2 and Class 3 populations showed a higher probability of sensitization to HDMs (Class 2 Der. p [100.0%], Der. f [99.38%] vs. Class 3 Der. p [100.0%], Der. f [100.0%]), and Class 3 has a high sensitized rate of animal dander (cat [100.0%], horse [62.07%], dog [48.28%]).

**FIGURE 4:**
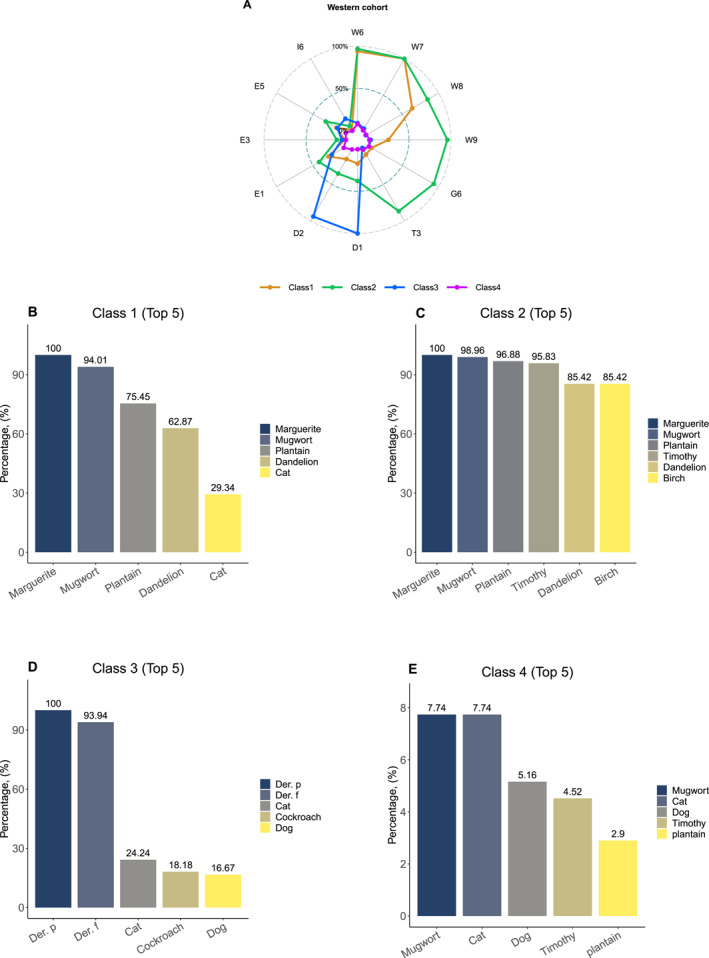
Differences in 12 aeroallergens sensitization patterns. (A) based on sIgE reactivity (B‐E) in the western cohort. In the western cohort, the radar plots showed the sensitization rate of each aeroallergen in different sensitization patterns with the posterior probability (Figure 4A). While the bar charts demonstrated the top 5 sensitization rates of 12 aeroallergens in different sensitization patterns (Figure 4B–E). 12 aeroallergens included W6 (mugwort), W7 (marguerite), W8 (dandelion), W9 (plantain), G6 (timothy), T3 (birch), D1 (*Dermatophagoides pteronyssinus*), D2 (*Dermatophagoides farinae*), E1 (cat), E3 (horse), E5 (dog) and I6 (*Blattella germanica*).

In the western cohort, 167 patients of Class 1 (26.14%) were labeled as having high sensitization to weed pollen. While 96 patients of Class 2 (15.02%) showed a high probability of sensitization to weed, tree, and grass pollens. High HDMs‐dominated sensitization was noted in 66 patients of Class 3 (10.33%) population, while low aeroallergen sensitization was observed in 310 patients of Class 4 (48.51%) (Figure 4A). Additionally, as shown in the Figure 4B–E, patients in Class 1 and Class 2 were characterized by high sensitization to pollens; the top 3 sensitization rates were marguerite, mugwort, and plantain, followed by dandelion and cat (Class 1)/birch (Class 2). Patients in Class 3 demonstrated a significantly high sensitized rate to HDMs primarily, while Class 4 populations presented a low probability of sensitization to all 12 aeroallergens.

### 3.4 Differences of age among distinct sensitization patterns

**FIGURE 5:**
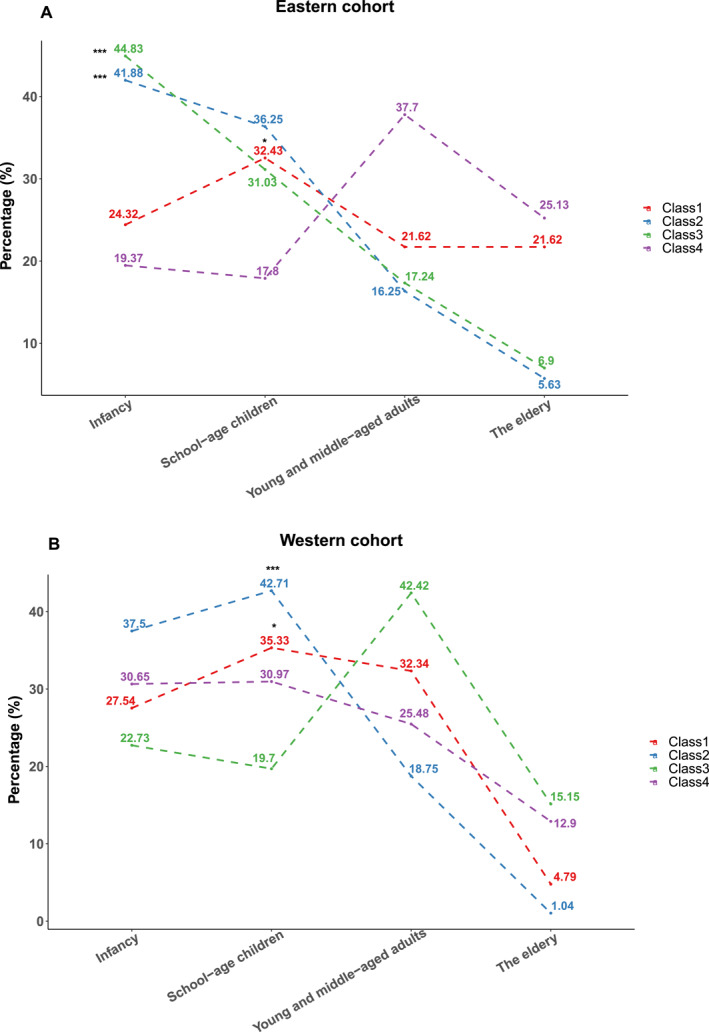
Comparisons of age among different sensitization patterns in eastern (A) and western (B) cohorts. In the eastern cohort, compared with the reference category (Class 4), results showed that asthma patients with HDMs‐dominated high sensitization, Class 2 (41.88% vs. 19.37%, *p* < 0.001) and Class 3 (44.83% vs. 19.37%, *p* < 0.001), were mainly infancy (aged 0–6 years). While the Class 1 population (32.43% vs. 17.80%, *p* < 0.05) showed a trend of sensitization during the school‐age children period (aged 7–14 years) (Figure 5A). In the western cohort, results revealed that the patients of Class 1 (35.33% vs. 30.97, *p* < 0.05) and Class 2 (42.17% vs. 30.97%, *p* < 0.001) groups tend to have high proportions of school‐age children (7–14 years) than other clusters (Figure 5B) (*, *p* < 0.05; **, *p* < 0.01; and ***, *p* < 0.001.).

Age played a critical role in different sensitization patterns. In the eastern cohort, based on the reference category (Class 4), results showed that asthma patients with high HDMs‐dominated sensitization, Class 2 (41.88% vs. 19.37%, *p* < 0.001) and Class 3 (44.83% vs. 19.37%, *p* < 0.001), were mainly aged 0–6 years, suggesting that infancy was susceptible to AA induced by HDMs (*p* < 0.001). Besides, HDMs‐sensitized asthma patients combined with pollen and animal dander sensitization (Class 1) showed a trend of sensitization during the school‐age children period, indicating the probability of poly‐sensitization was increased with rising age and exposure to various allergens (32.43% vs. 17.80%, *p* < 0.05) (Figure 5A).

In the western cohort, results revealed that the patients of Class 1 (35.33% vs. 30.97, *p* < 0.05) and Class 2 (42.17% vs. 30.97%, *p* < 0.001) groups tend to have high proportions of school‐age children (7–14 years) than other clusters. In contrast with different cohorts with similar sensitization characteristics, patients predominantly with high sensitization to HDMs focused more on young and middle‐aged adults in the western cohort population (Class 3, 42.42%, *p* < 0.05), while patients in the eastern cohort mainly aged 0–6 years (Class 2, 41.88%, *p* < 0.01). Differences in age proportion between the western and eastern cohort cohorts showed statistical significance (≤6 years, 22.73% vs. 41.88%, *p* = 0.006; 14<age≤50, 42.42% vs. 16.25%, *p* = 0.019), implying the occurrence of AA induced by HDMs in the patients of the western cohort probably later than that in the eastern populations (Figure 5B).

### 3.5 Differences in ACT score and comorbidities among different sensitization patterns

**FIGURE 6:**
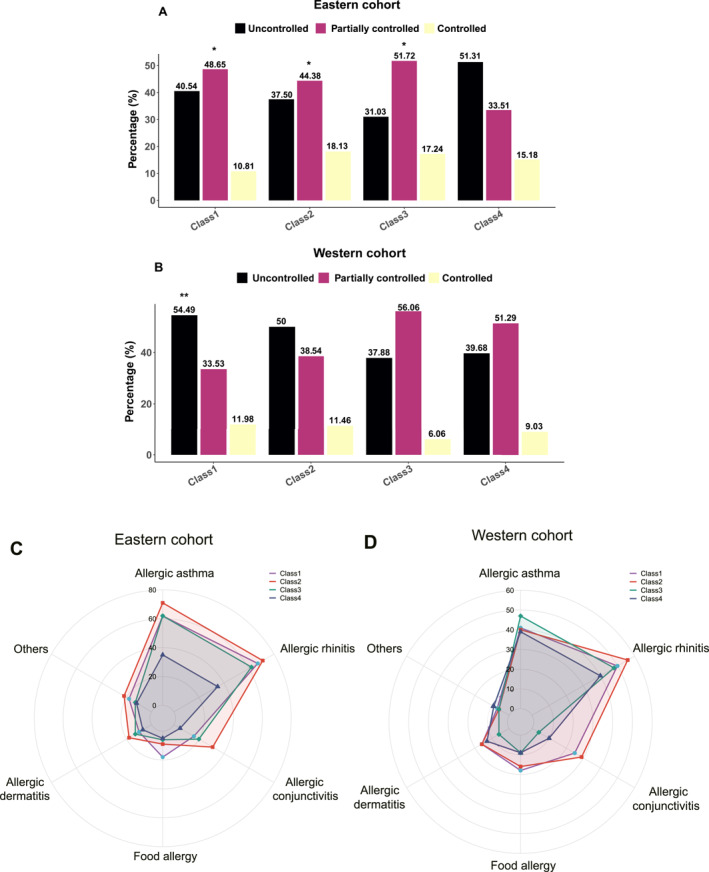
Comparisons of ACT score (A, B) and comorbidities (C, D) among different sensitization patterns. In the eastern cohort, compared with the reference category (Class 4), the proportion of ACT scores ranging from 20 to 25 was higher in Class 1, 2 and 3 populations. (Class 1–4: 48.65% vs. 44.38% vs. 51.72% vs. 33.51%, *p* < 0.05) (Figure 6A). In the western regions, in comparison with Class 4 populations, results showed that the ACT score of patients in Class 1 (ACT score <20, 39.68% vs. 54.49%, *p* < 0.01) mainly ranged less than 20 (Figure 6B). In the eastern cohort, compared with other groups, the radar plot manifested that asthma patients in the Class 1 population showed a higher incidence of food allergy (FA) (18.92%, *p* < 0.01), while the remarkably high sensitization to house dust mites (HDMs) asthma patients was vulnerable to suffering from Allergic conjunctivitis (AC) (Class 2 [31.88%], Class 3 [20.69%], *p* < 0.05) (Fig. 6C). While in the western cohort, half of the patients with asthma were combined with Allergic rhinitis (AR) at the same time (Class 1, 50.90%; Class 2, 56.25%; Class 3, 48.48%). In addition, patients with pollens and animal dander sensitization were prone to suffering from AC (Class 1, 25.75%; Class 2 29.17%; *p* < 0.05), while high HDMs‐sensitized populations were apt to getting food allergies (Class 3, 9.09%, *p* < 0.01) (Figure 6D) (*, *p* < 0.05; **, *p* < 0.01; and ***, *p* < 0.001.).

In the eastern cohort, ACT results demonstrated that asthma patients' scores mainly ranged from 20 to 25, except for the Class 4 population (33.51%, *p* < 0.05). Concerning the Class 4 population, the non‐atopic asthma patients' phenotype may account for the low ACT score, showing a relatively low sensitization (<5.0%) to these 12 aeroallergens (Figure 6A).

In the western regions, firstly, in comparison with Class 4, the ACT score of patients in Class 1 (ACT score<20, 39.68% vs. 54.49%, *p* < 0.01), with relatively high sensitization to pollens, mainly ranged less than 20, indicating that patients sensitized to both pollen and animal dander had a poor prognosis. Moreover, the difference between the patients with similar sensitization patterns in the two cohorts was discovered. Patients with high sensitization to HDMs in the eastern cohort (Class 2) showed a relatively poor prognosis than in the western cohort populations (Class 3). Compared with patients with polysensitization patterns (Class 1 and Class 2), patients with monosensitization patterns (Class 3) presented more patients with an ACT score ranging between 20 and 25 (i.e., asthma was partially controlled). According to GINA criteria, the higher the ACT score, the better control of the symptoms of asthma, demonstrating that monosensitization patterns may be more effective in asthma control and treatments in comparison with the polysensitization patterns (Figure 6B).

Meanwhile, in the eastern cohort, over 60.0% of patients with asthma suffered from Allergic rhinitis (AR) (Class 1, 67.57%; Class 2, 71.25%; Class 3, 62.07%). Interestingly, compared with other categories, we found that patients in Class 1, weed pollen and HDMs co‐sensitization, showed a higher incidence of food allergy (18.92%, *p* < 0.01), while the high sensitization to HDMs asthma patients were vulnerable to suffering from Allergic conjunctivitis (AC) (Class 2 [31.88%], Class 3 [20.69%], *p* < 0.05). In the western cohort, half of the asthma patients coexisted with AR (Class 1, 50.90%; Class 2, 56.25%; Class 3, 48.48%). In addition, patients in Class 1 and Class 2 were prone to suffering from AC (Class 1, 25.75%; Class 2 29.17%; *p* < 0.05), while high HDMs‐sensitized populations were apt to get food allergies (Class 3, 9.09%, *p* < 0.01) (Figure 6C,D). Characteristics of distinct patterns of aeroallergen sensitization among different cohorts were summarized in Table 2.

**TABLE 2:** Characteristics of sensitization patterns in different cohorts.

Cohort	Class	Sensitization patterns	Percentage (%)	Characteristics		
Eastern cohort	Class 1	High weed pollen and HDMs sensitization	8.87	Aged 7–14 years	Partially controlled	AR, AS, FA
	Class 2	HDMs dominated sensitization	38.38	Aged 0–6 years	Partially controlled	AR, AS, AC
	Class 3	High HDMs and animal dander sensitization	6.95	Aged 0–6 years	Partially controlled	AR, AS, AC
	Class 4	Low/no aeroallergen sensitization	45.80	Aged 15–50 years	Uncontrolled	AR, AS, others
Western cohort	Class 1	High weed pollen sensitization	26.14	Aged 7–14 years	Uncontrolled	AR, AS, AC
	Class 2	High multi‐pollen sensitization	15.02	Aged 7–14 years	Uncontrolled	AR, AS, AC
	Class 3	High HDMs‐dominated sensitization	10.33	Aged 15–50 years	Partially controlled	AR, AS, FA
	Class 4	Low/no aeroallergen sensitization	48.51	Aged 7–14 years	Partially controlled	AR, AS, Urticaria

Abbreviations: AC: allergic conjunctivitis; AR: allergic rhinitis; AS: allergic asthma; FA: food allergy.

### 3.6 Multiple logistic regression analysis

**FIGURE 7:**
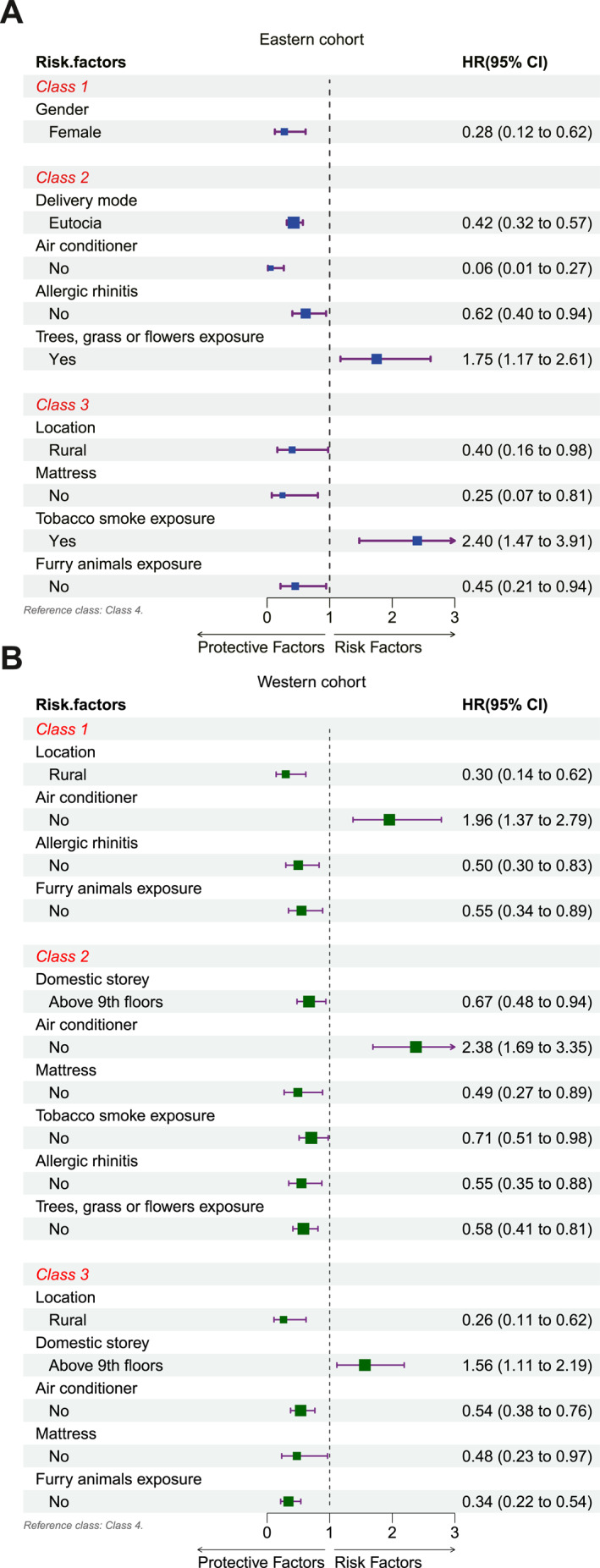
Risk factors were revealed by multiple logistic regression analysis. In the eastern cohort, compared with Class 4 (reference category), females in Class 1 presented as a protective factor. While in Class 2, natural labor, without Allergic rhinitis (AR) and not using air conditioners are protective factors, but pollen exposure could be a risk factor. In Class 3 populations, living in rural areas or not using mattresses or without furry animal exposure are protective factors, while tobacco smoke exposure is a risk factor (Figure 7A). In the western cohort, compared with the Class 4 (reference category), living in the suburbs, without AR and furry animal exposure are protective factors in Class 1, whereas do not use air conditioners is a risk factor. In Class 2 populations, not using air conditioners is a risk factor, while without AR, tobacco smoke and pollen exposure, not using the mattress, and living in a high domestic storey (≥9 floors) are protective factors. On the contrary, living in a high domestic storey (≥9 floors) is a risk factor in Class 3 populations. While living in the countryside, without furry animal exposure, not using air conditioners and mattresses are protective factors (Figure 7B).

In the eastern cohort, compared with Class 4, females in Class 1 presented relatively low sensitization to aeroallergens than males (OR 0.28, (95% CI 0.12–0.62), *p* = 0.002). While in Class 2, patients with these protective characteristics, including natural labor (OR 0.42, (95% CI 0.32–0.57), *p* = 0.001), without AR (OR 0.62, (95% CI 0.40–0.94), *p* = 0.026) and not using air conditioners (OR 0.06, (95% CI 0.01–0.27), *p* = 0.001), showed a low tendency of sensitization to aeroallergens, but patients with pollen exposure could be a risk factor (OR 1.75, (95% CI 1.17–2.61), *p* = 0.006). In Class 3 populations, patients living in rural areas (OR 0.40, (95% CI 0.16–0.98) or not using mattresses (OR 0.25, (95% CI 0.07–0.81), *p* = 0.021) or without furry animal exposure (OR 0.45, (95% CI 0.21–0.94), *p* = 0.035) may decrease the risk of sensitization. As an independent risk factor, tobacco smoke exposure (OR 2.40, (95% CI 1.47–3.91), *p* = 0.001) will predispose patients to sensitization (Figure 7A).

In the western cohort, compared with the Class 4 populations, patients who live in the suburbs (OR 0.30, (95% CI 0.14–0.62), without AR (OR 0.50, (95% CI 0.30–0.83), *p* = 0.008) and furry animal exposure (OR 0.55 (95% CI 0.34–0.89), *p* = 0.014) seems to decrease the risk of sensitization to aeroallergens in Class 1, whereas do not use air conditioners (OR 1.96, (95% CI 1.37–2.79), *p* = 0.001) may be a risk factor. A similar risk factor presented in Class 2 populations, those who rarely use air conditioners were easier to sensitization to aeroallergens (OR 2.38, (95% CI 1.69–3.35), *p* = 0.001), while patients without AR (OR 0.55, (95% CI 0.35–0.88), *p* = 0.012), without tobacco smoke (OR 0.71, (95% CI 0.51–0.98), *p* = 0.036) and pollen (OR 0.58, (95% CI 0.41–0.81), *p* = 0.002) exposure, do not use mattress (OR 0.49, (95% CI 0.27–0.89), *p* = 0.018), live in high domestic storeys (≥9 floors) (OR 0.67, (95% CI 0.48–0.94), *p* = 0.020) showed a low probability of sensitization. On the contrary, as a risk factor, living in a high domestic storey (≥9 floors) was observed in Class 3 populations (OR 1.56, (95% CI 1.11–2.19), *p* = 0.010). Patients with high HDMs‐dominated sensitization tend not to sensitize with the characteristics, including living in the countryside (OR 0.26, (95% CI 0.11–0.62), *p* = 0.002), without furry animal exposure (OR 0.34, (95% CI 0.22–0.54), *p* = 0.001), do not use air conditioners (OR 0.54, (95% CI 0.38–0.76), *p* = 0.001) and mattress (OR 0.48, (95% CI 0.23–0.97), *p* = 0.040) (Figure 7B).

## 4 DISCUSSIONS

To our knowledge, it is the first large‐scale multi‐center cross‐sectional study to characterize the sensitization profiles of aeroallergens assessed on asthmatic patients from two different geographical locations respectively, covering 10 medical centers situated in 10 provinces in mainland China. Over the past decade, asthma affected millions of people worldwide, and terribly, the number of patients will be up to 400 million by 2025.^11^ Our results showed a roaring trend of asthmatic patients (66.95%) and higher polysensitization rates (44.70%, Additional File 1 Table S4), in conformity with the growing allergic population in recent studies.^12^

Latent class analysis, a well‐validated statistical modeling program, was widely used to identify the phenotypes in various allergic respiratory diseases recently, revealing the potential associations and mechanisms between diseases and phenotypes.^13^, ^14^ Traditional hierarchical clustering analysis has some inherent shortcomings. Taking the most commonly used K‐means clustering analysis as an example, it has the following shortcomings: (a) The K‐means algorithm randomly selects the initial clustering center, which cannot guarantee the quality of the clustering effect. (b) The value of K should be determined in advance, and there is a large error in the artificial decision. (c) When the amount of data is insufficient, the different order of input data will lead to different results. (d) It is impossible to determine which attribute contributes more to clustering results. In addition, the traditional clustering analysis methods are mainly based on PCA. The essence of PCA is to reduce the number of variables, explain as much information as possible with a few variables, and maintain the independence of the obtained principal component through orthogonal transformation, which inevitably leads to missing information that cannot be explained. In terms of purpose and function, LCA can be regarded as a combination of PCA and cluster analysis. Its essence is to search for potential common factors (i.e., latent variables). Compared with traditional cluster analysis, LCA has the following advantages: (a) There is no need to eliminate variables to ensure the integrity of information reflected by explicit variables. (b) LCA can model a given number of categories and compare to get the most appropriate model, reducing the error caused by artificially specified K value. (c) LCA has nothing to do with the order of input data and the mutual order of data variables. Besides, we also analyzed the patients' data using both HCA and LCA. After comparing the cluster results of two different methods and taking the patient's medical records and questionnaires into consideration, we decided to adopt the LCA classification results which were more consistent with the patients' real situation (clinical manifestations, ACT score, subjective feelings, etc.). Although LCA and machine learning can both be used for data analysis and modeling, they differ significantly in terms of methods, objectives, and applications. Latent class analysis emphasizes the discovery of latent structures and patterns in data, while machine learning is more focused on the use of algorithms to achieve task automation and optimization. Therefore, both LCA and machine learning have their unique advantages and suitable application contexts, and it cannot be simply stated that one can replace the other. In this study, in addition to LCA, we also used machine learning algorithms, random forest analysis, for subsequent data analysis and verification (Figure 7). This allowed us to explore possible risk factors in different cluster populations, providing patients with potential risk warnings and avoiding risk factors in advance.

Our findings indicated that asthma patients with sensitization to multiple respiratory allergens could be clustered and summarized into four distinct sensitization profiles. Of the four sensitization patterns, three clusters were labeled as moderate or high sensitization to HDMs, which widely prevailed in the eastern region. It is well known that HDMs, as the most prevalent aeroallergen, sensitized over 90% of atopic patients in Asia.^15^ Compared with a recent national cross‐sectional study conducted from 2008 to 2018, HDMs were still the most popular aeroallergens in China.^16^ In our study, HDMs and pollens sensitization was observed in nearly half of the cases of AA patients in the eastern and western regions, respectively, consistent with Lebanese asthmatic patients.^17^ Similarly, relevant results were observed in Southeast Asia and Asia areas, reporting a high sensitization rate to HDMs in the Philippines (97.4%),^18^ Singapore (68.5%),^19^ Thailand (65.0%),^20^ and South Korea (21.4%).^21^ Moreover, in comparison with the former study (13.38%),^22^ a slight increase in the sensitization rate of HDMs (20.0%) in western regions, indicating that more importance should be attached to co‐sensitization to mites when doctors diagnose allergy patients in western areas.

While in the western cohort population, more complicated sensitized profiles were discovered, demonstrating a sensitization to weed, tree and grass pollens or HDMs. Compared with patients with similar sensitization patterns in the western cohort, asthmatic patients of Class 1 in the eastern cohort manifested a somewhat higher proportion in ACT scores ranged 20–25. Even though this finding nearly reached statistical significance overall (*p* = 0.05) in Pearson's chi‐squared analysis, the Fisher exact test calculated a significant statistical difference (*p* = 0.033). Besides, evidence was shown in some patients' medical records in this sampling, presenting relatively severe clinical symptoms like wheezing, dyspnea and chest pain in the eastern cohort. The limited number of enrolled participants may account for the insignificant statistical difference. Besides, a group of non‐allergic asthma patients characterized by “Low/no aeroallergen sensitization” were poorly controlled in the eastern. However, debates still existed about the differences in disease severity between atopy and non‐atopy asthma patients. Some reported that allergic asthmatic children tended to have better clinical scores and milder symptoms than non‐allergic children,^23^ while others argued that atopy or not was not associated with patients' poor control of asthma.^24^

With regards to age, plenty of researchers have reported that the probability of poly‐sensitization was increased with rising age and exposure to various allergens.^12^ As is known, infancy was susceptible to AA induced by HDMs. Similar results were observed in preceding studies conducted in Qatar,^25^ Iran^26^ and Saudi Arabia,^27^ manifesting that HDMs sensitization was more common in early childhood. Possibly, the more time spent indoors, the more exposure to indoor allergens. Staying at home and keeping away from outdoor activities was a usual phenomenon in response to the lockdown policy during the COVID‐19 pandemic. That might be one of the causative reasons for younger children having a more prevalent sensitization to HDMs than adolescents and adults. Interestingly, a phenomenon was noted that the occurrence of AA triggered by HDMs in the patients of the eastern cohort probably earlier than that in the western populations in our study, suggesting that children living in eastern areas of China, especially coastal cities, should raise people's awareness of sensitization to HDMs earlier.

As shown herein, the notable coexistence between AC and asthma was observed in patients sensitized to HDMs from eastern regions (Class 2 and Class 3) or pollens from western cohorts (Class 1 and Class 2) in our study. Consistent with preceding researches, patients allergic to mites^28^ and pollens^29^ showed a tendency of suffering from AC more frequently, indicating that sensitization to mites or pollens may be a risk factor in atopy diseases. Food allergy was noticed in asthmatic patients sensitized to pollens in eastern regions (Class 1), presenting a relatively high prevalence than in other categories. Pollen‐food allergy syndrome may account for a substantial part of the reasons for some fruit, vegetable and nut allergies in pollen‐sensitized patients due to cross‐reactivities among profilin^30^, ^31^. Asthma patients with the sensitization patterns mentioned above should pay more attention to the relevant comorbidities in the early stage of the disease.

In conclusion, several limitations of this study should be pointed out. Firstly, it is a retrospective study we cannot acquire the complete clinical characteristics of all patients. Thus, some valuable test results related to sensitization, like blood routine examination, imaging examination, pulmonary function test and so on, could be missed and ignored possibly. Secondly, some confounding factors were difficult to eliminate, owing to a lack of detailed baseline information, for instance, the progression of allergic disease and patients' physical condition at the beginning. Third, only one thousand participants who met all criteria were enrolled in our research approximately, for the reason that quite a large part of patients were lost to follow‐up during the COVID‐19 pandemic. Furthermore, about half of the individuals included were non‐allergic asthma patients, which might blur some of the sensitized characteristics of the general atopic asthma population. Additionally, the mechanisms of several exposure factors varying in different sensitization patterns remained unknown. The risk ratios of exposure failed to calculate because we could not prove the causative correlation between exposure and outcomes based on the cross‐sectional study design. Finally, aimed to identify sensitization patterns by using the most cost‐effective allergens combo, the inhaled allergens panel merely consists of 12 representative allergens in mainland China, which might lead to deviation in patients clustering based on their sensitization profiles.

Despite the limitation of the cross‐sectional study, it is noted that participants enrolled in our study were of substantial representativeness in general asthmatic patients in China. If possible, additional prospective research will probably be better to determine and verify whether these latent classes conform with corresponding asthma patients clinically. Therefore, further studies need to be explored to reveal the underlying mechanism and association of atopic respiratory diseases, providing novel viewpoints and possible strategies for precision diagnosis and prevention in the future.

## 5 CONCLUSIONS

In summary, based on the levels of sIgE reactivity, our findings indicated that asthma patients with sensitization to multiple respiratory allergens could be clustered and summarized into four distinct sensitization profiles through the LCA statistical modeling method. Hence, focusing on the western and eastern areas, this multicenter study successfully recognized and unveiled the differences between sensitization patterns, geographical differences and clinical characteristics in asthmatic patients.

## AUTHOR CONTRIBUTIONS

Baoqing Sun and Chuangli Hao conceived the study and revised the manuscript. Jiale Zhang, Wenting Luo, Guoping Li, Huali Ren, Jie Su, Jianxin Sun, Ruifen Zhong, Siqin Wang, Zhen’an Li, Yan Zhao, Huashou Ke, Ting Chen, Chun Xv, Liting Wu, Xianhui Zheng, Zhenglin Chang, Miaoyuan Xv and Qingyuan Ye conducted experiments and participated in the data acquisition. Jiale Zhang, Wenting Luo, Liting Wu and Xianhui Zheng completed statistical analysis and visualization. Jiale Zhang and WL drafted the manuscript. All authors read and approved the final manuscript.

## CONFLICT OF INTEREST STATEMENT

The authors declare that they have no competing interests.

## CONSENT FOR PUBLICATION

All authors reviewed the manuscript and revised it critically. All authors approved the final version of the manuscript.

## Supporting information

Supporting Information S1

## Data Availability

The data that support the findings of this study are available on request from the corresponding author.
