# Supplementary material for: Patterns of aeroallergen sensitization in asthma patients identified by latent class analysis: A cross‐sectional study in China
Source: Clin Transl Allergy. 2023 Jul 1;13(7):e12271. doi: 10.1002/clt2.12271 (PMC10314277; doi:10.1002/clt2.12271)
Supplement: Supplementary file 1 — Supporting Information S1 [file CLT2-13-e12271-s001.docx]

**Supporting information**

**Table S1. Latent class analysis model fit comparison (Eastern cohort)**

| Model | AIC | BIC | aBIC | Entropy | LMR p-value | BLRT *p*-value |
| --- | --- | --- | --- | --- | --- | --- |
| 3-Classes | 2623.595 | 2776.853 | 2656.268 | 0.969 | 0.105 | < 0.001 |
| 4-Classes | 2536.500 | 2742.187 | 2580.351 | 0.965 | < 0.001 | < 0.001 |
| 5-Classes | 2499.037 | 2757.155 | 2554.066 | 0.966 | 0.1103 | < 0.001 |

*Note: AIC = Akaike Information Criterion; BIC = Bayesian Information Criterion; aBIC = Adjusted BIC; LMR = Vuong-Lo-Mendell-Rubin Likelihood RatioVuong-Lo-Mendell-Rubin Likelihood Ratio; BLRT = Parametric Bootstrapped Likelihood Ratio Test.*

**Table S2. Latent class analysis model fit comparison (Western cohort)**

| Model | AIC | BIC | aBIC | Entropy | LMR p-value | BLRT *p*-value |
| --- | --- | --- | --- | --- | --- | --- |
| 3-Classes | 5525.512 | 5694.989 | 5574.342 | 0.964 | < 0.001 | < 0.001 |
| 4-Classes | 5272.875 | 5500.330 | 5338.409 | 0.965 | < 0.001 | < 0.001 |
| 5-Classes | 5263.538 | 5548.972 | 5345.777 | 0.961 | 0.101 | < 0.001 |

*Note: AIC = Akaike Information Criterion; BIC = Bayesian Information Criterion; aBIC = Adjusted BIC; LMR = Vuong-Lo-Mendell-Rubin Likelihood RatioVuong-Lo-Mendell-Rubin Likelihood Ratio; BLRT = Parametric Bootstrapped Likelihood Ratio Test.*

**Table S3. Prevalence of asthma alone and asthma combined with rhinitis in different age**

| **Region** | **Age (yr)** | **Asthma alone (N, %)** | **Asthma combined with rhinitis (N, %)** |
| --- | --- | --- | --- |
| **Eastern cohort (N=417)** | ≤6 | 11 (2.64) | 115 (27.58) |
|  | 6＜x≤14 | 7 (1.68) | 105 (25.18) |
|  | 14＜x≤50 | 17 (4.08) | 95 (22.78) |
|  | ＞50 | 26 (6.23) | 41 (9.83) |
|  | Total | 61 (14.63) | 356 (85.37) |
|  |  |  |  |
| **Western cohort (N=639)** | ≤6 | 36 (5.63) | 155 (24.26) |
|  | 6＜x≤14 | 32 (5.01) | 179 (28.01) |
|  | 14＜x≤50 | 39 (6.10) | 139 (21.75) |
|  | ＞50 | 21 (3.29) | 38 (5.95) |
|  | Total | 128 (20.03) | 511 (79.97) |

**Table S4. Monosensitization and polysensitization rates of 12 aeroallergens in different age**

| **Region** | **Age (yr)** | **Non-sensitization (N, %)** | **Monosensitization (N, %)** | **Polysensitization (N, %)** |
| --- | --- | --- | --- | --- |
| **Eastern cohort (N=417)** | ≤6 | 42 (10.07) | 5 (1.20) | 79 (18.94) |
|  | 6＜x≤14 | 15 (3.60) | 0 | 97 (23.26) |
|  | 14＜x≤50 | 63 (15.11) | 6 (1.44) | 43 (10.31) |
|  | ＞50 | 49 (11.75) | 3 (0.72) | 15 (3.60) |
|  | Total | 169 (40.53) | 14 (3.35) | 234 (56.12) |
|  |  |  |  |  |
| **Western cohort (N=639)** | ≤6 | 64 (10.02) | 19 (2.97) | 108 (16.90) |
|  | 6＜x≤14 | 56 (8.76) | 14 (2.19) | 141 (22.07) |
|  | 14＜x≤50 | 66 (10.33) | 15 (2.35) | 97 (15.18) |
|  | ＞50 | 42 (6.57) | 4 (0.63) | 13 (2.03) |
|  | Total | 228 (35.68) | 52 (8.14) | 359 (56.18) |
